# Application of 3D bioprinting in the prevention and the therapy for human diseases

**DOI:** 10.1038/s41392-021-00566-8

**Published:** 2021-05-14

**Authors:** Hee-Gyeong Yi, Hyeonji Kim, Junyoung Kwon, Yeong-Jin Choi, Jinah Jang, Dong-Woo Cho

**Affiliations:** 1 Department of Rural and Biosystems Engineering, College of Agriculture and Life Sciences, Chonnam National University, 77 Yongbong-Ro, Gwangju, 61186 Korea; 2 Department of Mechanical Engineering, Pohang University of Science and Technology (POSTECH), 77 Cheongam-Ro, Pohang, Kyungbuk 37673 Korea; 3 Department of Advanced Biomaterials Research, Korea Institute of Materials Science (KIMS), 797 Changwondaero, Changwon, Kyungnam 51508 Korea; 4 Department of Convergence IT Engineering, POSTECH, 77 Cheongam-Ro, Pohang, Kyungbuk 37673 Korea; 5 Institute of Convergence Science, Yonsei University, 50 Yonsei-Ro, Seoul, 03722 Korea

**Keywords:** Translational research, Tissue engineering

## Abstract

Rapid development of vaccines and therapeutics is necessary to tackle the emergence of new pathogens and infectious diseases. To speed up the drug discovery process, the conventional development pipeline can be retooled by introducing advanced in vitro models as alternatives to conventional infectious disease models and by employing advanced technology for the production of medicine and cell/drug delivery systems. In this regard, layer-by-layer construction with a 3D bioprinting system or other technologies provides a beneficial method for developing highly biomimetic and reliable in vitro models for infectious disease research. In addition, the high flexibility and versatility of 3D bioprinting offer advantages in the effective production of vaccines, therapeutics, and relevant delivery systems. Herein, we discuss the potential of 3D bioprinting technologies for the control of infectious diseases. We also suggest that 3D bioprinting in infectious disease research and drug development could be a significant platform technology for the rapid and automated production of tissue/organ models and medicines in the near future.

## Introduction

**Fig. 1:**
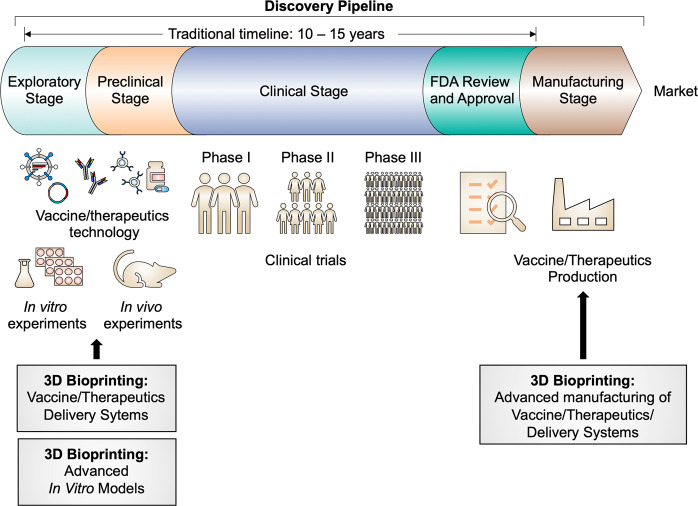
Schematic drawing of the traditional vaccine/therapeutics discovery pipeline and the possibilities of retooling with 3D bioprinting technologies (gray boxes)

The emergence of a new pathogen can cause unexpected diseases and epidemics in humans, livestock, and wildlife. Until 2002, coronaviruses were known to cause a common cold in humans.^1^ However, since then, new coronavirus mutations (e.g., SARS-CoV and MERS-CoV) have caused severe respiratory syndromes and have become life-threatening pathogens for humans. Moreover, coronaviruses continuously evolve, which has resulted in the development of a virus with high transmission, causing the current global coronavirus disease 2019 (COVID-19) pandemic.^2^ The current pandemic is creating breaks in interactions and networks in human societies and causes serious economic crises worldwide.^3^ The primary principle of infection control is the prevention of the spread of the disease. In this respect, the COVID-19 pandemic has taught that a global imperative need is a platform to implement the agile and rapid development of vaccines and therapeutics against the emergence of a new pathogen, such as SARS-CoV-2.^4–7^ However, the traditional drug development process typically takes 10–15 years to develop not only an effective but also a safe drug.^8^ Therefore, retooling the drug discovery pipeline via emerging sophisticated biofabrication technologies (e.g., nano- and micro-fabrication, soft lithography for creating microfluidic devices, and 3D bioprinting) has been gaining interest for the acceleration of drug discovery pipelines (Fig. 1).

In the exploratory and preclinical stages of drug discovery (Fig. 1), the refinement of infectious disease models can reduce trial and error. Biofabrication aims to manufacture biological systems or therapeutic products by integrating cells, biomaterials, and biomolecules.^9^ As biofabrication technologies facilitate the creation of a desired living construct, they have been adopted for engineering an advanced in vitro model: a miniaturized living tissue or organ that recapitulates physiological features within an in vitro setting.^10,11^ In the development of advanced in vitro models, reverse engineering is the process of extracting essential information from a living tissue, organ, or body system to identify the minimal set of design principles required to reproduce a new analog.^12^ In this process, biofabrication contributes to building a highly biomimetic structure containing the desired arrangement of multiple cell types within an engineered context. Unlike conventional in vitro cell cultures, advanced in vitro models have been demonstrated to reproduce the physiology of native tissues and even the pathological characteristics of diseases.^10,11^ In addition, the advanced in vitro model is advantageous for modeling species-specific diseases and treatment responses by directly using target species-derived cell sources.^13^ Collectively, the advanced in vitro model takes advantage of higher similarities to native tissues and reproducibility than conventional in vitro and in vivo models. In this regard, the biofabrication of the advanced in vitro model is expected to be a significant tool to overcome the challenges of reducing the time taken to identify an effective candidate in the drug discovery process.

The production of drugs and drug delivery systems also accounts for a significant portion of the pipeline (Fig. 1). The ease of producing vaccines/therapeutics is a key criterion for accelerating the drug development-production process.^8^ In line with this, biofabrication technology can support the manufacture of vaccines and therapeutics. As a biofabrication approach allows the integration of various cells, biomaterials, and other molecules with therapeutic and/or functional properties, this technology can facilitate the easy fabrication of advanced but complex therapeutic products.^14,15^ Nano-biofabrication technology has formulated effective nanomedicine supported by the development of DNA/RNA/protein engineering technologies.^16^ In addition, nano- and micro-biofabrication technologies have reinforced the delivery efficiencies of therapeutic cells and molecules with beneficial biomaterials.^17,18^ Delivery systems have been established in various forms according to the delivery route, for example, injection,^15^ oral administration,^19^ and implantation.^20^ Taken together, biofabrication technologies have contributed to improvements in both the engineering and production of medicine and delivery systems. Therefore, biofabrication is a promising approach for advancing the current vaccine/therapeutic development process.

Notably, 3D bioprinting has emerged as a core technology for biofabrication. 3D bioprinting has a distinctive characteristic compared to other biofabrication technologies owing to its principle of layer-by-layer construction combined with spatial patterning of multiple types of materials on demand. Recently, 3D bioprinting has been advanced to utilize cells as building blocks to construct living tissues and organs from the ground directly.^21,22^ The encapsulated live cells in pre-gel solutions have been utilized as bioinks to 3D print the desired tissue structures.^23^ Therefore, 3D bioprinting has recently begun to be used for engineering advanced in vitro models as well.^24–26^ The 3D bioprinting of in vitro models has shown that it is advantageous for creating entire systems containing living tissues and microfluidic architecture in a one-step fabrication process through sequences of multi-material printing.^27^ Moreover, 3D bioprinting stands out as being highly flexible and versatile in producing various therapeutic products because of the wide range of applicable materials.^14^ 3D bioprinted drugs have been established by depositing therapeutic molecules as oral tablet^28^ or in implantable form.^20^ In particular, 3D bioprinting has strength in the customization of medicine by combining different types of therapeutic materials at various doses according to personalized condition.^29^ Overall, 3D bioprinting has the potential to advance both in vitro models and vaccine/therapeutics engineering for better control of infectious diseases.

In this article, we review the recent applications of bioprinting, other biofabrication technologies in engineering in vitro models, and vaccine/therapeutics production for the drug development pipeline against infectious diseases. In particular, we discuss 3D cell-printing technologies that directly employ live cell-laden hydrogels to build a 3D tissue construct. First, we briefly describe bioprinting technologies and relevant materials. We then review the in vitro models created with various biofabrication technologies for infectious disease research and introduce 3D bioprinted in vitro models that have the potential to be applied as infection disease models. Next, we cover the recent vaccine/therapeutics technologies and 3D bioprinting techniques for producing drugs and delivery systems applicable to infectious diseases. Finally, we briefly discuss the current challenges and future perspectives of 3D bioprinting-based approaches to confronting the emergence of new pathogens and epidemic diseases.

## Advances in 3D bioprinting

In recent years, considerable attention has been focused on the utilization of 3D bioprinting for building functional tissues, organs, and therapeutics.^22^ 3D bioprinting allows the control of biomaterials, biomolecules, and cells in a layer-by-layer process to create a 3D tissue construct with geometrical complexities.^30^

Here, we summarize the materials currently utilized for 3D bioprinting inks and advanced 3D bioprinting technologies for producing in vitro models and therapeutic systems.

### Advanced materials for 3D bioprinting

In bioprinting, bioink is mainly used as a biocompatible hydrogel and is utilized to protect cells from damage that would occur during the printing process.^21,26^ In addition, the bioink is geometrical support for the 3D structure. To mimic the hierarchical architecture of native tissue, most studies have focused on improving the printability, viscosity, rapid cross-linking, and mechanical properties of the bioink.^31^ Bioink has begun to be regarded as a microenvironmental niche that provides bioactive cues (e.g., extracellular matrix (ECM), growth factor, and binding site) to the residing cells and induces tissue formation and maturation.^32^

In recent tissue engineering investigations, the materials used in 3D bioprinting of tissues, organs, and therapeutic products are categorized into (1) biomaterial inks and (2) bioinks, depending on whether cells are encapsulated.^33^ In this section, we discuss biomaterial inks and hydrogels applicable to formulate bioink.

#### Biomaterial inks

The frameworks should physically support 3D in vitro models and retain the various shapes of each batch. To print the framework, the biomaterial ink should have good printability without clogging and flexible versatility in each printing condition. Polycaprolactone (PCL),^34,35^ polydimethylsiloxane (PDMS),^36,37^ and their derivatives can provide physical and mechanical support to in vitro models with little influence on the cells or cellular behaviors based on their biocompatibility.

In particular, PCL, an FDA-approved biomaterial, has been widely used as a drug delivery carrier in sutures, and as scaffolds for tissue repair due to its long-term stability and slow biodegradability.^38^ However, because PCL is hydrophobic, cells hardly attach to their surface. Therefore, the improvement of PCL has been studied to enhance its bioactivity through surface functionalization or by preparing its composites.

PDMS is a silicone-based organic compound and is the most popular in engineering organ-on-a-chip with soft lithography.^39^ PDMS is transparent, non-toxic, and non-flammable; therefore, it has been widely used in medical devices. In particular, as cured PDMS shows rubber-like high flexibility in solid-state, soft lithography with PDMS has led to the construction of a micro-sized structure in a transparent device.^40^ In the process of soft lithography, a mold containing a micropattern is constructed through lithography using a laser, and then PDMS is applied for casting the pattern. In this process, the superior flexibility of PDMS resulted in the construction of channels on the microscale to manipulate an extremely small amount of fluidic flow. Therefore, soft lithography with PDMS has evolved to create an organ-on-a-chip with the precise control of fluids and localization of specific cells to the desired position.^41^ In addition, 3D bioprinting with PDMS has been attempted to construct a transparent and stable chip device, while cells are bioprinted inside the PDMS chip.^13,42^

Poly(ethylene glycol) (PEG) has been widely used as a biocompatible ink because of its high tunability and affinity for biomolecules.^43–47^ PEG is water-soluble and, thus, has been used as a sacrificial material for complex and hollow-shaped frameworks. To improve cellular interactions, conjugated PEG can be made with biomimetic ligands, such as short peptide sequences or larger proteins, including drugs. Modified or conjugated PEG (e.g., poly(ethylene glycol) diacrylate (PEGDA), polyethylene glycol dimethacrylate) can then be used as a cell-encapsulating ink.^48,49^ The modified PEG provides a cell adhesion site, facilitates high bioactivity, improves protein adsorption and covalent coupling with cell-adhesive peptide sequences, and enhances tunable stiffness.

#### Hydrogels applicable to bioink

Hydrogels provide a cell-friendly matrix to recapitulate native ECM microenvironments because of their tunable physical properties, biodegradability, and bioactive functions.^21^ Cells or biochemical molecules can be encapsulated in hydrogels to promote tissue regeneration and reconstruction. Hydrogels for bioink are required to (1) flow under modest pressures, (2) solidify quickly, and (3) sustain adequate integrity after building up.^50,51^ In the sol–gel transition process, fibers in solutions can be physically or chemically cross-linked by external stimuli, such as temperature, light source, or ion concentration.^52^ The main strength of physical cross-linking is the absence of cytotoxic chemical agents. On the other hand, chemical cross-linking occurs through the covalent bond formation. Thus, the resulting hydrogel exhibited excellent mechanical properties. However, chemically cross-linked hydrogels usually undergo more volume changes than physically cross-linked hydrogels. Collectively, the number of hydrogels applicable to bioinks is limited, and adjusting the physical/chemical properties is still difficult.

Natural source-derived hydrogels have been widely used as bioinks, of which there are many types: alginate,^53,54^ collagen,^55,56^ gelatin,^57,58^ cellulose,^59^ silk fibroin,^60,61^ and decellularized ECM.^23,62–64^ Alginate is derived from brown algae and is a low-cost, biodegradable, and cytocompatible material. Alginate can be cross-linked by simple immersion in a CaCl_2_ solution.^53,65^ In particular, their mechanical properties, including tensile strength, Young’s modulus, and elongation, can be regulated by the concentration of the cross-linker calcium chloride. However, alginate does not provide binding sites for mammalian cells; therefore, human cells cannot experience cell–ECM interactions and form an appropriate morphology. Thus, modified alginate, such as the addition of Arg-Gly-Asp (RGD) or gelatin, has helped improve cell attachment.^65^

Collagen, the most abundant component in mammalian body systems, has been promoted as a pivotal alternative for bioinert bioink.^56^ Because of the ubiquitous nature of these proteins, they can be used across a variety of species without an immunogenic response. Collagen showed low mechanical properties after thermal cross-linking, but a rapid degradation rate. To enhance its mechanical properties, collagen has been hybridized with natural molecules (e.g., glycosaminoglycans, tricalcium phosphates) and synthetic polymers (e.g., polyglycerol methacrylate).

Gelatin is a water-soluble protein and a denatured form of collagen. As it retains the RGD sequence from collagen, gelatin promotes cell adhesion and proliferation.^58^ Although gelatin dissolves as a colloidal sol at physiological temperatures (37 °C), it can form a gel when the temperature drops to <29 °C. To overcome these reversal trends with collagen, many attempts, including blending with other materials, chemical modifications (e.g., methacrylated gelatin and biomolecule-conjugated gelatin), and additive cross-linkers, have been reported.

Cellulose, a biologically derived polysaccharide biopolymer, is an important structural component of the cell walls of plants.^59^ Porous cellulose hydrogels possess good transparency and desirable mechanical stability. Thus, this material can be utilized as a drug carrier to deliver pharmaceutical agents, contact lenses, or wound healing materials.

Silk fibroin, usually obtained from the silkworm *Bombyx mori*, has been established as a suitable biomaterial for the culture of different types of cells, including those from breast cancer,^66^ prostate cancer,^67^ osteosarcoma,^68^ and hepatocellular carcinomas.^69^ Silk fibroin hydrogels have good biocompatibility and processability; however, they must be further engineered to either encourage normal tissue regeneration or enhance tumorigenicity and the malignant performance of cancer cells.^70,71^

Finally, ECM-based hydrogels have emerged as promising materials. Matrigel is derived from a basement membrane composite secreted by Engelbreth-Holm-Swarm mouse sarcoma cells.^72^ Therefore, Matrigel is suitable to promote tumorigenic growth and invasion and is thus extensively used for the culture of tumor cells. On the other hand, decellularized ECM (dECM) has been presented as a novel tissue-specific ECM material that emulates the native composition of a tissue matrix.^73^ Decellularization is the process of removing the residing cells and remaining the ECM from tissues and organs. The high efficiency of decellularization can provide tissue-specific ECM compositions obtained from various livestock. As dECM contains a large portion of collagen, dECM can be solubilized through pepsin-mediated digestion. The solubilized dECM showed cross-linking properties at physiologically relevant pH values and temperatures. Above all, since recapitulating the native environment of each tissue, dECM hydrogels have promoted cellular activities compared with single-element-containing hydrogels, such as collagen. However, some limitations in printability have been reported and attempts to improve the mechanical properties have recently been carried out.^74,75^

### 3D bioprinting technologies

A variety of 3D bioprinting techniques have been developed and utilized to create highly functional 3D structures, including extrusion-based, light-assisted, and inkjet-based printing systems. These systems commonly produce 3D structures with high fidelity through computer-aided design and computer-aided manufacturing.^76^ Each bioprinting system has a different resolution and unique dimensions for printing each layer. Plane-by-plane bioprinting was performed with digital light processing (DLP). In the case of extrusion-based bioprinting, line-by-line bioprinting was performed. Finally, point-by-point bioprinting has been demonstrated in laser-induced forward transfer (LIFT), stereolithography (SLA), and inkjet-based bioprinting.

#### Extrusion-based bioprinting

**Fig. 2:**
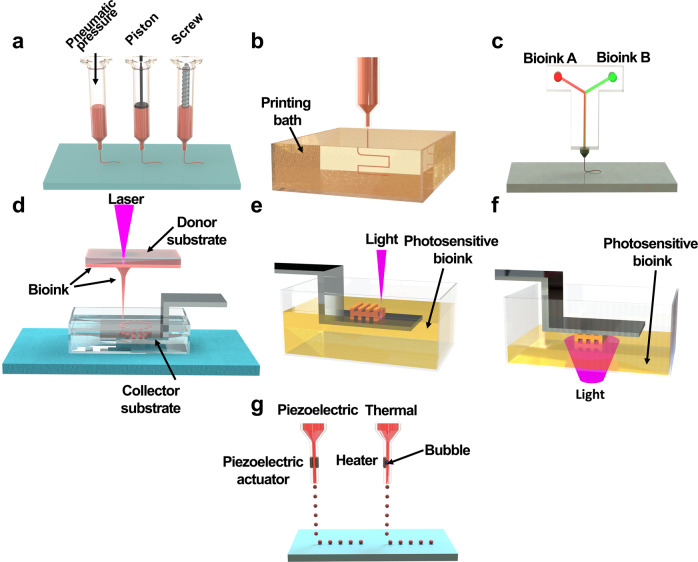
Schematic diagrams of 3D bioprinting technologies. **a** Extrusion-based bioprinting, **b** FRESH-based bioprinting, **c** microfluidic chip-assisted bioprinting, **d** laser-induced forward transfer (LIFT), **e** stereolithography (STL), **f** digital light processing (DLP), **g** inkjet-based bioprinting

Extrusion is the most widely used 3D bioprinting method. Extrusion-based bioprinting dispenses the cells containing bioink through a nozzle by applying various pressures driven by air, screws, and a piston (Fig. 2a).^77^ The dispensed bioink is placed spatiotemporally in a 3D pattern through the adjustable movement of the printing head in the *X*-, *Y*-, and *Z*-axes. The resolution of extrusion-based printing can be controlled by adjusting the nozzle size, feed rate (printing speed), and pressure. The printing resolution is approximately 200 μm, which is relatively low compared to other bioprinting techniques.^78^ Because this method is based on extrusion, shear stress can occur as the bioink passes through the nozzle. As shear stress can cause cell damage and death, high cell viability can be achieved by carefully controlling the parameters (pressure, bioink viscosity, nozzle size, and shape) of the inducible shear stress.

Bioink deposition is required to build a 3D tissue construct for extrusion-based printing. However, most of the hydrogels used in bioink formulations have low viscosity, making material deposition difficult. To overcome these limitations, recent studies have utilized specific modules to improve the deposition ability of bioinks. A coaxial nozzle can supply both bioink and cross-linking buffer simultaneously to achieve 3D bioink deposition (alginate-CaCl_2_).^79^ A ultraviolet (UV) curing module is installed in the print head to cure photocurable bioinks as soon as they are dispensed from the nozzle (GELMA).^57^ In the case of collagen bioink, a thermal module is installed in the print head to induce thermal cross-linking of the collagen bioink to promote 3D structure stacking.^80^

In addition to using these various modules, a technology that can promote the effective deposition of low-viscosity bioinks has recently been developed. The freeform reversible embedding of suspended hydrogels (FRESH)-based printing technique can perform 3D bioprinting and cross-link at the same time by dispensing low viscosity bioink into a granular gel bath (Fig. 2b).^81^ This technology allows the direct production of 3D tissue structures using low-viscosity bioinks, thereby expanding the availability of various bioinks. Recently, this technology has been applied to various fabrication applications, such as for muscles, the heart, and blood vessels.^63,82,83^ However, as the FRESH technique primarily depends on the cross-linking mediated by the chemical reaction between the bioink and the bath material, the selection of materials is relatively narrow.

To build a 3D complex tissue structure with multiple cells using extrusion-based printing technology, bioprinting must be conducted by placing bioinks containing different cells on multiple print heads. Multiple printing heads can produce heterogeneous 3D tissue constructs by alternately discharging different bioinks. As the tissue becomes more complex, the number of cell types, bioinks, and printing heads increases, resulting in a dramatic increase in printing time, which can affect cell viability. Recently, microfluidic chips have been applied to the dispensing head, allowing a variety of bioinks to be dispensed in one nozzle (Fig. 2c).^84^ This technology facilitates the rapid fabrication of complex and heterogeneous 3D tissue structures.

#### Light-assisted printing

**Fig. 3:**
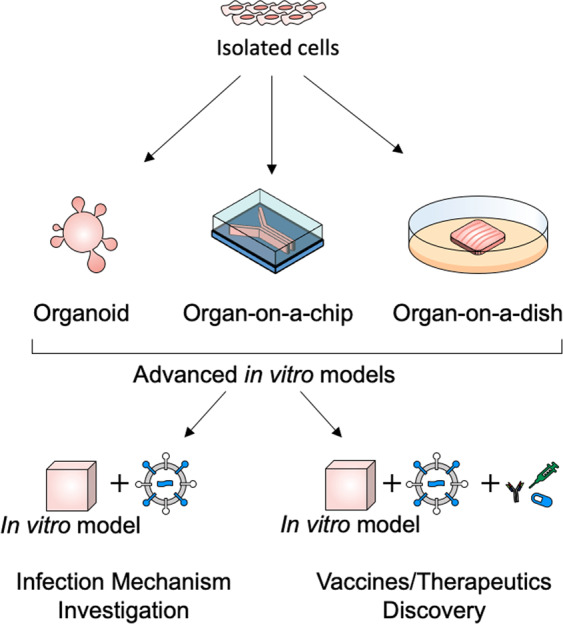
Advanced in vitro models used in research of infectious disease to identify the infection mechanisms and the effective vaccines/therapeutics

Light-assisted printing is a bioprinting technique that uses light to solidify a photocurable bioink. Light-assisted printing can be divided into three types: (1) LIFT (Fig. 2d), (2) SLA (Fig. 2e), and (3) DLP (Fig. 2f).

The LIFT bioprinting apparatus consists of three main constituents: (1) a pulsed laser source, (2) a donor substrate that acts as a support for the bioink, and (3) a collector substrate for the bioink.^85^ The donor substrate is composed of glass or quartz, which does not absorb the laser, and gold or titanium. Bioinks containing cells are prepared by spreading them onto the surface of a donor substrate. When laser pulses are exposed to the donor substrate, droplets are formed in the bioink and fall onto the collector substrate. The collector substrate is movable along the *X*- and *Y*-axes, and the bioink droplets are continuously stacked on the collector substrate to produce a 3D shape. Bioink droplets deposited on the collector substrate require an additional cross-linking process. For LIFT bioprinting of alginate bioinks, a CaCl_2_ bath was added to the collector substrate to induce cross-linking of the alginate bioink droplets.^86^ LIFT bioprinting does not require the use of nozzles and photocurable bioinks, and 3D structures can be precisely fabricated using bioinks in the low viscosity range (1–300 mPa s). However, LIFT bioprinting technology has the disadvantage of creating a volumetric structure because it requires coating a donor substrate of a 1 m^2^ bioink film when fabricating a 1 cm^3^ structure.^86^

SLA bioprinting is a representative light-assisted printing method in which photocurable bioinks are subjected to UV, infrared, or visible light to create 3D structures through the layer-by-layer method. SLA induces the selective curing of photocurable bioinks point-by-point, while DLP technology uses a computer-controlled dynamic mirror array to selectively project and cross-link the bioink at the 2D layer level simultaneously. Therefore, 3D tissue structure fabrication through DLP is much faster than that through SLA. These printing methods facilitate the fabrication of cell patterns of complex geometries with sub-micrometer resolution. Owing to these advantages, they have recently been widely applied to tissue fabrication that requires the implementation of microstructures, such as perfusable blood vessels and capillaries.^87,88^

While the existing light-assisted printing techniques are slow because these approaches fabricate 3D tissue constructs in units of points or layers, the recently developed volumetric printing method is considerably faster (from several to tens of seconds).^89^ The volumetric printing method uses three overlapped laser beams to create a holographic pattern and selectively cure the photocurable bioink in the bath to produce a 3D tissue structure. As the three laser beams are used at the same time, the time taken to cure the bioink is greatly reduced, thereby overcoming the limitations of the existing one-layer stacking manufacturing method.

#### Inkjet-based printing

Inkjet-based printing (Fig. 2g), known as the first bioprinting study, uses a modified office inkjet printer to distribute cells and biomaterials to generate 2D living cell patterns.^90^ This bioprinting method can generate and accurately place picoliter volumes (1–100 pL) of bioink onto a substrate. Each bioink droplet can be generated through two inkjet printing strategies: (1) continuous or (2) drop-on-demand inkjet printing. The size of the bioink droplet of continuous inkjet printing ranges from 10 to 150 μm, and the droplets are electrically conductive and can be placed and stacked on a substrate by an electric or magnetic field. Bioink droplets created by drop-on-demand inkjet printing can be generated by thermal, piezoelectric, or acoustic approaches.^91–93^ Drop-on-demand inkjet printing can selectively generate bioink droplets on the substrate, and the resulting droplets show high resolution in the <30 μm range. Continuous inkjet printing produces droplets much faster than drop-on-demand inkjet printing systems; however, owing to the requirements for conductive fluid ink and the risk of contamination in the process, the drop-on-demand inkjet printing method is widely used for 3D tissue structure fabrication. The droplet size and deposition rate depend on the viscosity and surface tension of the bioink, and the control of the printing path and droplet deposition process can be adjusted by changing the voltage and pulse duration. While inkjet-based bioprinting enables the creation of a high-resolution, precise 3D tissue structure at a relatively low cost, the limited range of bioink viscosity (1–200 mPa s) and difficulty in building volumetric 3D tissue constructs are considered major drawbacks.^94^

## Biofabricated in vitro models for studying infectious diseases

The lack of experimental models has hindered the rapid unraveling of the pathogenesis of infection and effective drugs. Animal models have contributed to the exploration of vaccines and therapeutics for infectious diseases, such as Salmonella infection,^95^ Ebola virus disease,^96^ and even COVID-19,^97^ animal models inherently cannot fully recapitulate the interactions between humans and pathogens, resulting in unsatisfactory reliability in testing experimental targets.^98^

Conventional in vitro human cell culture platforms, such as a monolayer culture of immortalized human cell lines, are cost-effective and convenient for screening experimental targets. However, as the conventional approach cannot reflect the complex and dynamic responses of human organs, models that can accurately reproduce the interactions between humans and pathogens are still lacking.^10^

Advanced in vitro models have been developed to contain human tissue and the relevant microenvironment, such as anatomical structures, body fluid flows, and simultaneous active mechanical and biochemical cues.^11,12^ Engineering in in vitro models is emerging to bridge the gap between conventional experimental models and human infectious diseases.^10,98^ Recently, microfluidic organs-on-chips have been adopted for the modeling of infectious diseases. However, because the employment of advanced in vitro tissue models in infectious disease research is still in its infancy, robust outputs have not yet been achieved in 3D bioprinting-based approaches. Therefore, this section covers comprehensive technologies for building in vitro human tissue models, including (1) organoids, (2) soft-lithography-based microfluidic organs-on-chips for studying infectious diseases, and (3) possibilities of 3D bioprinting in modeling infectious diseases (Fig. 3).

### Organoids

Organoids have been developed to morphologically and functionally reproduce human organs consisting of multiple types of cells. Due to this advantage, in recent years, human organoids have been robustly leveraged to identify the pathology following either bacterial^99,100^ or viral infection.^101^ Human proximal airway organoids have demonstrated its ability to discriminate human-infective influenza virus (H7N9) from poorly human-infecting viruses, such as avian-infective influenza virus (H7N2) and swine-infective influenza virus (H1N1).^102^ In addition, human infant lung organoids have been developed to simulate a respiratory virus infection that commonly occurs in infants and children.^103,104^ In particular, human organoids have quickly been utilized to confront the challenge of developing an effective treatment for COVID-19. Studies regarding SARS-CoV-2 infection in human organoids of the eyes,^105^ airway,^106^ liver,^107^ intestines,^108^ kidneys,^109^ and brain^110,111^ have identified how SARS-CoV-2 induces damage to many types of organs. In the human eye organoid, the limbus is most infected by the virus, whereas the central cornea is less susceptible to infection.^105^ In human airway organoids, the virus mainly infects and replicates in the basal epithelial cells,^106^ which co-express *ACE2* and *TMPRSS2*, which are major portals for the infection and transmission of SARS-CoV-2,^112^ while the virus is not detected in other cell types, such as cilia and club cells. In addition, a human liver organoid has demonstrated that SARS-CoV-2 infection disrupts the transport function of cholangiocytes and the barrier function of bile ductal epithelium,^107^ implying liver damage, which is a common feature in patients with severe cases of COVID-19. A human intestinal organoid exhibited significant production of infective SARS-CoV-2 particles, suggesting a fecal-oral transmission route.^108^

### Microfluidic organs-on-chips

Microfluidic organ-on-a-chip recapitulates tissue-tissue interactions, microfluidic flows, and dynamic mechanical motions to capture native-like physiology. Microfluidic organs-on-chips have been employed to study infectious diseases in many organ types.

**Fig. 4:**
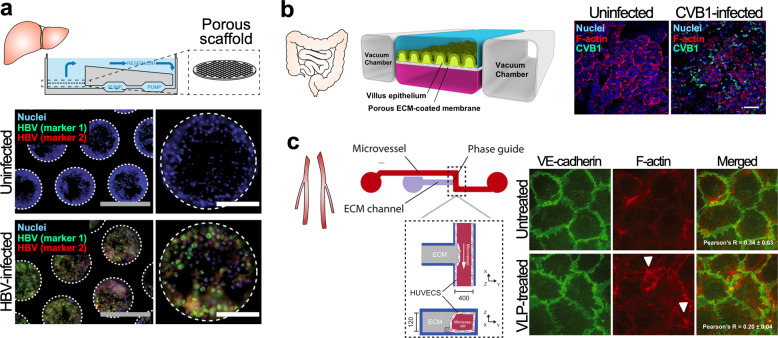
Microfluidic-based human organs-on-chips applied in the study of infectious diseases. **a** Liver-on-a-chip for the study of hepatitis B virus (HBV) infection. Adapted from Ortega-Prieto et al.^113^ **b** Gut-on-a-chip for the study of Coxsackie B1 Virus (CVB1) infection. Adapted from Villenave et al.^115^ **c** Vascular endothelium barrier-on-a-chip for evaluating the effect of Ebola virus-like particle (VLP) on vascular integrity. White arrows indicate remodeling of F-actin. Adapted from Junaid et al.^116^

A human liver-on-a-chip to model hepatocyte organization on liver sinusoids with a microfluidic recirculation system was used to study hepatitis B virus (HBV) infection^113,114^ (Fig. 4a). As the liver-on-a-chip is permissive to long-term observation for more than 40 days, the chip recapitulates all steps of the HBV lifecycle from replicating the virus to the maintenance of covalently closed circular DNA (cccDNA). Following HBV infection, the liver-on-a-chip recapitulated the recognition failure of HBV by Kupffer cells, stellate macrophages of the liver, and lower secretion of IL-6 and TNF-α, suggesting the suppression of innate immune activation.

A human gut-on-a-chip was utilized to capture the polarized infection of the Coxsackie B1 (CVB1) virus^115^ (Fig. 4b). The gut-on-a-chip was established to form villus intestinal epithelium on a porous ECM-coated membrane under dynamic conditions with continuous fluid flow and peristalsis-like periodic strains. Villenave et al. detected CVB1 infection from the cell apex of the gut-on-a-chip following the introduction of the virus into either the epithelial lumen located at the upper channel or the basal channel located at the lower by separation with the porous ECM-coated membrane of the device. This polarized infection of the virus also corresponded to the higher secretion of inflammatory cytokines, such as IP-10 and IL-8, from the epithelium lumen, compared to the basal channel.

Furthermore, a human vascular endothelium barrier-on-a-chip was employed to reproduce disruption of vascular integrity and hemorrhagic shock syndrome following Ebola virus infection^116^ (Fig. 4c). The chip allowed the formation of a vascular endothelium barrier along the microchannel, and an Ebola virus-like particle (VLP) was introduced into the endothelial lumen. The VLP-treated lumen showed cytoskeleton remodeling with an increase in F-actin and a higher permeability than the VLP-untreated lumen. In addition, similar phenomena were observed following treatment with U46619, an activator of the Rho/Rock pathway. Subsequently, treatment with Ebola glycoprotein also induced an increase in actin filament stress fibers and the permeability of the endothelium barrier. Taken together, as the chip demonstrated the modulation of Rho/Rock by Ebola viral infection, an experimental drug, FX06, and melatonin were tested and showed efficacy on the chip.

Si et al.^117^ utilized a human lung-on-a-chip to repurpose clinically available drugs as therapeutics for influenza and COVID-19. The lung-on-a-chip has double-layered channels divided by a porous membrane to mimic the structure of the alveolus. In this system, human epithelial cells are grown on the middle membrane and exposed to continuous airflow, while human vascular endothelial cells are attached to the opposite side of the membrane and exposed to continuous liquid flow. The dynamic air–liquid interface promotes the differentiation of human airway epithelial cells to highly express the genes *ACE2* and *TMPRSS2*. Moreover, the lung-on-a-chip suggested that only two drugs, amodiaquine, and toremifene, had significant effects on viral entry reduction among the seven approved drugs pre-screened as inhibitors of SARS-CoV-2 S protein infection using a conventional culture of Huh-7 cell lines,^118^ which are widely used for viral infection screening in vitro. Although the relevance to clinical observation has not yet been unveiled, the investigation by Si et al. has demonstrated how an in vitro model can be adopted for a fast track to potential therapeutics for new infection pandemics.

### Possibilities of 3D bioprinting in modeling infectious diseases in vitro

In addition to the abovementioned research, 3D bioprinting is an emerging technology in tissue engineering. Soft lithography, the most widely applied method for creating organs-on-chips, has exhibited remarkable results in the construction of 3D organs and tissues through the micro-manipulation of fluids. However, as soft lithography is based on sequential assemblies of the pre-cast parts, the freedom of 3D construction of bioprinting is relatively higher than that of the assembly-dependent method. Layer-by-layer construction maximizes design flexibility, which is required for engineering tissues.

Many researchers have developed 3D-bioprinted functional tissues, which have enormous potential as screening systems (Table 1). In this section, we discuss 3D-bioprinted tissues and screening systems in the following five categories: (1) tissues in contact with foreign substances (such as the cornea and airway); (2) the nervous system; (3) the circulatory system; (4) other homeostatic systems (e.g., lungs, liver, kidneys, and pancreas); and (5) the high-throughput screening system.

**Table 1:** 3D bioprinting for engineering tissues and its application to high-throughput screening platforms

Target organ	Printing method	Bioink	Cell type	Ref.
*Engineered tissue*				
Skin	Inkjet	N/A	Primary human epidermal keratinocytes	^119^
	Extrusion	Skin-derived ECM with fibrinogen	Human dermal fibroblast	
	Extrusion	Adipose-derived ECM with fibrinogen	Preadipocyte	
	Extrusion	Thrombin‐embedded 10% gelatin	Human umbilical vein endothelial cells (HUVECs)	
Cornea	Extrusion	Cornea-derived ECM	Pre-differentiated keratocytes	^64,120^
Airway	Stereolithography	Tracheal mucosa-derived ECM (tmdECM)	N/A	^121^
Airway-on-a-chip	Extrusion	tmdECM	Endothelial cells or fibroblasts	^122^
Nervous system	Micro-extrusion	N/A	Hippocampal neurons, the superior cervical ganglia neurons, and the peripheral nerve components using Schwann cells and epithelial cells.	^123^
Central nerve	Extrusion	N/A	Induced pluripotent stem cell‐derived spinal neuronal progenitor cells and oligodendrocyte progenitor cells	^124^
Vessel	Extrusion	Vascular-derived ECM with alginate	HUVEC	^125^
Vessel network	Extrusion	Gelatin, fibrinogen with transglutaminase	Human neonatal dermal fibroblasts, and human bone marrow-derived mesenchymal stem cells	^42^
Liver	Extrusion	Collagen	HepG2	^27^
		Gelatin	HUVEC	
Lung	Stereolithography	PEGDA	N/A	^87^
Pancreas	Extrusion	Pancreas-derived ECM	Human islet	^129^
Kidney	Extrusion	Gelatin, fibrinogen with transglutaminase and calcium chloride	Human neonatal dermal fibroblasts	^130^
	Extrusion	Gelatin, fibrinogen with transglutaminase and calcium chloride	N/A	^131^
	Extrusion	Kidney-derived ECM	Renal tubular epithelial and endothelial cells	^132^
*High-throughput screening platform*				
Intestine	Extrusion	N/A	Human placental-derived mesenchymal stem cells (h-PMSC), U87 MG human glioblastoma cells (U87), or human intestinal smooth muscle cells (h-ISMC)	^133^
Liver	Inkjet	N/A	HepG2	^134^
		N/A	HUVECs	

#### 3D-bioprinted tissues in contact with foreign substances

**Fig. 5:**
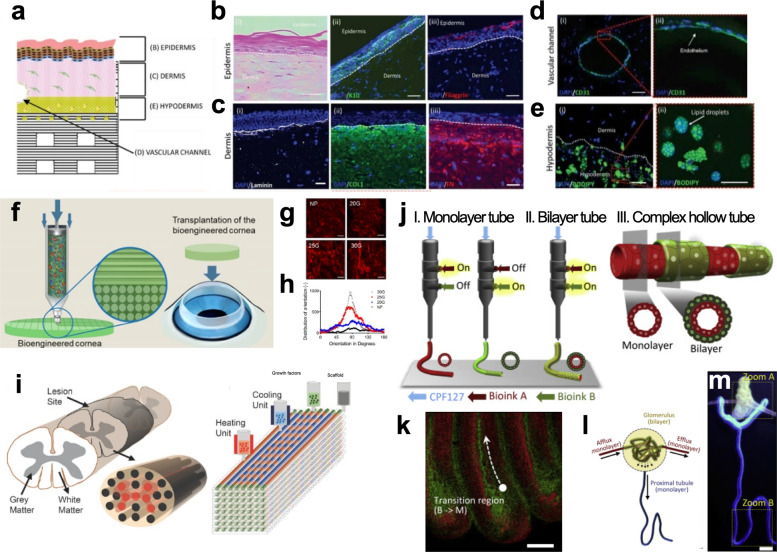
3D bioprinted in vitro models of human tissues and organs. **a** 3D cell-printed skin model composed of epidermis, dermis, hypodermis, and vascular channel. Stained images using representative markers of each layer: **b** epidermis, **c** dermis, **d** vascular channel, and **e** hypodermis (scale bars, 50 µm). Reproduced with permission from Kim et al.^119^ **f** 3D bioprinting of transparent corneal tissue via the alignment of collagen fibers within the nozzle during bioink extrusion. **g** Second-harmonic generation (SHG) images of shear-aligned collagen using each nozzle. (scale bar, 20 μm). **h** Distributions of collagen orientations at different azimuthal angles. Reproduced with permission from Kim et al.^120^ **i** Schematic diagram of the spinal cord illustrating gray matter and white matter boundaries and the 3D bioprinting process. Reproduced with permission from Joung et al.^124^ **j** Coaxial printing of monolayer and bilayer structures in complex hollow tubes. The schematic represents monolayer (I), bilayer (II), and fine-tuning between monolayer to bilayer at defined intervals in the complex hollow (III) tubes for renal tubular tissue. **k** A convoluted hollow tube with a transitional region between monolayer and bilayer structures. (scale bars, 1 mm). **l** Schematic representation of the glomerulus and proximal tubule in native kidney tissue. **m** 3D bioprinting of complex renal tubular structures. (scale bar, 500 μm). Reproduced with permission from Singh et al.^132^

Tissues in contact with foreign substances have specific structural characteristics. The skin has a papillary layer, which plays a role in regulating body temperature via capillaries within each papilla. Kim et al.^119^ 3D-printed a perfusable vascularized human skin equivalent composed of an epidermis (primary human epidermal keratinocytes), dermis (human dermal fibroblast‐encapsulated skin-derived ECM with fibrinogen), and hypodermis (preadipocyte‐embedded adipose-derived ECM with fibrinogen; human umbilical vein endothelial cell (HUVEC)–thrombin-containing 10% gelatin for vascularization) (Fig. 5a–e). They reported that this full-thickness skin reflects the actual complexity of native human skin more realistically than existing dermal and epidermal skin models. In addition, the vascularized dermal and hypodermal compartments improved the promotion of cross‐talk with the epidermal compartment, producing better recapitulation of epidermal morphogenesis.

The cornea has patterned lamellae in the stromal layer. The inner collagenous pattern of the corneal stroma affects its transparency. Kim et al.^64^ fabricated transparent corneal stromal tissue using cornea-derived decellularized ECM and shear stress upon the 3D cell-printing process^120^ (Fig. 5f, h). The printed structure recapitulated the native corneal macrostructure with aligned collagen fibrils, resulting in the construction of a highly mature and transparent corneal stroma equivalent. After 4 weeks of implantation in vivo, the collagen fibrils generated a lattice pattern similar to that of the human cornea, becoming more transparent.

With regard to the airway, the trachea is a hollow cylindrical organ that prevents the collapse of the tracheal lumen during respiratory movements, including rotation, flexion, and extension. Park et al.^121^ presented tissue-engineered tracheal grafts composed of a bellows framework with a tracheal mucosa decellularized ECM (tmdECM) using a DLP-bioprinting system. The tracheal grafts were transplanted to circumferential tracheal defects, and their lumens were maintained against any movement. The tmdECM-assisted grafts accelerated epithelial regeneration compared to the collagen-assisted control group, leading to complete epithelialization of the entire luminal surface 2 months postoperatively.

The tmdECM was then applied to develop a 3D-printed airway-on-a-chip where each endothelial cell or fibroblast-encapsulated tmdECM was deposited in the PCL frame.^122^ Endothelial cells were re-orientated in the 3D printed airway model and formed a lumen and blood vessel network. The human airway epithelium assembled with the vascular platform showed an improvement in tight junctional connections compared to the epithelium chip.

Taken together, 3D bioprinting demonstrated that this approach was able to build tissues in contact with foreign substances, such as the skin, cornea, and airway. These bioprinted tissues have shown high similarities with native tissues and, thus, have great potential for application in testing the direct infection of pathogens, barrier functions of the tissues, and the effectiveness of treatments.

#### 3D bioprinted nervous system

The nervous system is a complex network of nerves and cells that facilitates communication to and from the brain and spinal cord as well as to various parts of the body. The brain and spinal cord are categorized into the central nervous system (CNS), while the rest of the nerves and ganglia comprise the peripheral nervous system (PNS). 3D bioprinting technology enables the precise fabrication of replicated designs of complex nerve networks with multiple specific cells as well as the provision of directionality for regenerating axons, thereby encouraging neural network formation. Johnson et al.^123^ fabricated a nervous system-on-a-chip for the study of viral infections in the nervous system. The nervous system-on-a-chip is compartmentalized into three chambers: (1) the CNS chamber, including hippocampal neurons; (2) the PNS chamber, containing the superior cervical ganglia neurons; and (3) the peripheral nerve components, using Schwann and epithelial cells. The alignment of the axonal networks between the chambers and the spatial organization of the cellular components was achieved using micro-extrusion-bioprinting strategies. After viral infection of the peripheral neuron cells in the PNS chamber, the Schwann cells and hippocampal neurons showed a restriction of viral uptake with 1.4 and 1.6 genomes per cell, respectively, indicating that Schwann cells transmitted a pseudorabies virus through the axon-to-cell pathway but appeared refractory to infection.

Joung et al.^124^ presented a bioengineered spinal cord via extrusion-bioprinting technology (Fig. 5i). Clusters of iPSC‐derived spinal neuronal progenitor cells and oligodendrocyte progenitor cells were placed in 150 µm-wide channels with 200 µm center‐to‐center spacing. On the third day of scaffold culture, progressive axon extension along the channel was observed. The activity of the bioprinted neuronal system was confirmed by physiological spontaneous calcium flux studies, showing that the fluorescent intensity of these neuronal networks increased in response to high levels of potassium and neurotransmitter glutamate.

#### 3D bioprinted circulatory system

In new drug development research, the circulatory system is one of the primary subjects to observe pharmaco-kinetic and dynamic characteristics and the interactions between disease, vascular pathology, and the development of drugs. Thus, 3D bioprinting of vessels that can ensure endothelium barrier function and solution perfusion is essential in engineering advanced in vitro models. Gao et al.^125^ developed perfusable in vitro vascular models using the coaxial cell printing technique and HUVEC‐encapsulated vascular-derived ECM/alginate hybrid bioink. Various designs with endothelium lining in the luminal wall were constructed by one-step fabrication. Following the maturation of the endothelium, directional angiogenesis was demonstrated in response to the stimulation of proangiogenic factors, such as VEGF and bFGF. The sprout of the neovessels was only observed on the side with angiogenic cues, while none of the cells migrated on the signal-absent side. In addition, it was verified that inflammatory responses, including the promoted adhesion of immune cells, were observed by airway inflammatory signals.

Kolesky et al.^42^ constructed perfusable 3D vascularized tissues using a multi-material 3D bioprinting method. The vessel structure was bioprinted with a Pluronic solution containing thrombin and cross-linked with nearby ECM materials, including gelatin, fibrinogen, transglutaminase, and cells (human neonatal dermal fibroblasts and human bone marrow-derived mesenchymal stem cells (hMSCs)). Thereafter, the printed fugitive vascular inks were removed by liquefaction at 4 °C, and the vascular network was constructed by injecting the HUVEC suspension into the empty vascular channel. These thick vascularized models demonstrated their functionalities, in that hMSCs were differentiated into osteogenic lineages in situ by perfusion with osteogenic growth factors, including BMP-2.

The 3D bioprinted perfusable vessels provided a platform for identifying how the infected cells or pathogens passed through tissue barriers and circulated the body system. In addition, in vitro circulation emulated how changes in infection-induced cytokines can damage the circulatory system.

#### Other 3D bioprinted homeostatic organs

Most body systems control the regulation of homeostasis. This section covers the rest of the organs, including the liver, lungs, pancreas, and kidneys. Lee et al.^27^ presented a one-step fabrication approach for constructing an organ-on-a-chip with 3D bioprinting technology. This fabrication method enabled the construction of various heterogeneous and complex designs and allowed the development of a spatially heterogeneous liver-on-a-chip. The housing and microfluidic channels of the chip were fabricated with the hydrophobic polymer PCL, while the inside-3D cellular parts were 3D printed using HepG2-laden collagen. For 2D cell monolayer formation, HUVEC-laden gelatin was used and then removed by liquefying the gelatin. The liver-on-a-chip showed higher hepatocyte viability and enhanced levels of albumin/urea secretion.

Norona et al.^126^ developed a mini liver construct consisting of human hepatocytes, stellate cells, and Kupffer cells, using a 3D cell-printing system.^127^ The hepatic construct showed approximately tenfold increased viability and albumin secretion, respectively, compared to conventional 2D cultured hepatocytes for two weeks. The 3D cell-printed hepatic construct recapitulated methotrexate-induced fibrogenesis, following the signals of injury.

Grigoryan et al.^128^ constructed perfusable vascularized vital organs, including the lungs and liver, via DLP-bioprinting. An alveolar model consisting of a perfusable airway system and a perfusable ensheathing vasculature system was bioprinted with PEGDA. Although human red blood cells (RBCs) were perfused under deoxygenated conditions, the oxygen saturation of RBCs increased with decreasing RBC flow rate by bidirectional flows within the vessel segment after numerous ventilation cycles. In the case of liver construction, a perfusable network-containing hydrogel carrier was designed to deliver hepatic aggregates in vivo. The perfusable carrier tissue exhibited higher integration with host tissue and better albumin promoter activity at 14 days post-transplantation in mice with chronic liver injuries, compared to the delivered single type of cells.

Kim et al.^129^ developed 3D pancreatic constructs formed from an islet-encapsulated pancreatic tissue-derived ECM bioink via micro-extrusion-based printing. The fabricated pancreatic tissue showed that over 60% of human islets survived after five days of culture, and insulin secretion levels increased with an increase in the concentration of glucose in the culture medium. Moreover, the construct enhanced pancreatic functions, such as elevated RNA levels of pancreas-specific genes.

With advancements in biofabrication methods for 3D hollow tissue, renal tissue modeling has shown considerable evolution. Homan et al.^130^ first reported a 3D-printed platform of a human renal proximal tubule (PT). Customized chips were fabricated by extrusion-based printing of perfusable tubules within ECMs, promoting epithelialization with improved phenotypical and functional properties. The mature epithelium was applied in a disease model, which could be disrupted by cyclosporine A, a nephrotoxin, in a dose-dependent manner. Lin et al.^131^ bioprinted a 3D vascularized PT model composed of an ECM and two types of perfusable tubes, one with a PT epithelium and the other with vascular endothelium. Albumin uptake and glucose reabsorption were observed in the mature PT model, and the effect of a glucose transport inhibitor was investigated in a disease model of hyperglycemia. In addition to in vitro renal models, Singh et al.^132^ developed a microfluidic tubular/vascular renal parenchyma, which could be applied to in vivo transplantation (Fig. 5j–m). Each tubular/vascular hollow tube was cell-printed using 3D coaxial nozzles with kidney-derived ECM bioinks encapsulating renal tubular epithelial and endothelial cells, respectively. They showed the potential of microfluidic renal tissue models in a vascularized renal PT-on-a-chip as well as PT graft for long-term renal subcapsular transplantation.

#### 3D bioprinted high-throughput screening system

As tissue engineering research continues, studies involving personalized medicine are increasing. 3D bioprinting technology enables versatile fabrication using multi-cell-laden materials, taking only a few hours, while imparting possibilities for high-throughput and low-cost fabrication of microfluidic devices. In this regard, many studies have involved the development of 3D-printed personalized high-throughput systems for biological applications, including drug screening. Boyer et al.^133^ introduced 96-well cell culture inserts containing 3D spheroidal microtissues. The inserts were designed with negative hemispherical spacing at the bottom to hold the cell-laden droplets. Thereafter, extrusion-based 3D printer-seeded media contained human placenta-derived MSCs, U87 MG human glioblastoma cells, or human intestinal smooth muscle cells. After printing for 72 h, each cell generated cellular clusters, forming single spheroids. These inserts allowed control of the diameter of the spheroids by regulating cell density and permitted the screening of bioactive agents.

Matsusaki et al.^134^ presented 440 microarrays of simplified liver tissue chips with multilayered and multiple types of cells using inkjet printing technology. The multi-well-based chips were printed rapidly, and the single layer of HegG2s sandwiched between HUVEC layers showed improvement in both albumin and CYP3A4 secretion. Although these studies are in the early stages of personalized medicine research, they have demonstrated that inkjet-bioprinted high-throughput screening systems have the potential for personalized bioassay platforms. Therefore, we conclude that it could be adopted for further innovative approaches in the field of bioengineering.

## 3D bioprinting in engineering vaccines and therapeutics

**Fig. 6:**
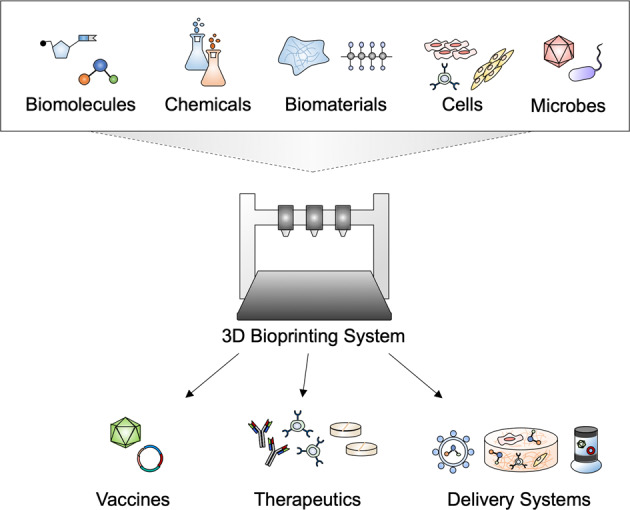
Applications of 3D bioprinting for manufacturing of vaccines, therapeutics, and delivery systems

The development of vaccines and therapeutics (e.g., antibiotics, antiviral drugs, antibodies, and therapeutic cells) still takes more than 10 years and three billion dollars on average for one new medicine to complete the process from initial discovery to reaching the marketplace.^8^ To address this pharmaceutical challenge, nano- and micro-technologies have been merged for the production of vaccines and therapeutics to accelerate the medicine development process and improve the delivery efficiency and efficacy of new medicines. This section describes the current vaccine platforms and therapeutics applied in the treatment of various infectious diseases and reviews the possibility of adapting 3D bioprinting technologies for producing vaccines, therapeutics, and delivery systems (Fig. 6).

### Vaccine technologies

This section briefly introduces the landscape of the modern vaccine platforms used in treating various infectious diseases prior to discussing the possibility of using 3D bioprinting in the development of vaccines and therapeutics.

Vaccines are biological substances that elicit the immune system to fight against infectious viruses and bacteria. Therefore, vaccination is currently the most successful medical approach for preventing and controlling infectious diseases. Typically, there are whole-pathogen vaccines, subunit/conjugate vaccines, and nucleic acid vaccines. In particular, the current vaccines under phase 3 clinical trials or FDA approval for COVID-19 are listed in Table 3 (updated on the 23rd of December 2020; adapted from WHO website^135^).

Attenuated or inactivated microbes are historical vaccines. Live-attenuated vaccines are designed to produce infections without (or with very weak) symptoms. For this purpose, scientists select particular weakened strains made by repeating the culture.^136^ The vaccine then generates an immune response similar to natural infection and confers long-term immunity without causing illness or spreading to other individuals. Several live-attenuated vaccines have been developed, such as MMR combined (measles, mumps, and rubella), rotavirus, smallpox, and yellow fever vaccines.^137,138^ Inactivated vaccines have been developed using pathogens that have lost their symptomatic characteristics through heat, radiation, or chemical treatments.^139^ These vaccines cannot provide as strong immunity as live-attenuated vaccines because of their inability to replicate in vivo. Thus, killed vaccines are generally less immunogenic than live vaccines. To compensate for this, killed vaccines are often administered together with an adjuvant (such as aluminum salts) to increase their potency or are injected two to three times for ongoing immunity.^140^ However, killed virus vaccines have fewer side effects than live virus vaccines. Several viral and bacterial vaccines are produced in this manner, such as those against influenza, hepatitis A, and rabies virus, as well as whole-cell *Bordetella pertussis*.^140,141^

Subunit vaccines only include specific antigens or components that stimulate the immune system instead of the whole pathogen. Specific antigens are used as components of vaccines, and recombinant proteins are also used as antigens, which are combined to elicit the best immune response.^142^ Although this design can make vaccines safer and easier to produce, it often requires the incorporation of adjuvants to elicit a strong protective immune response because the antigens alone are not sufficient to induce appropriate long-term immunity. On the other hand, it includes only the essential parts for boosting the immune system, as the vaccine can minimize side effects. Unlike protein-based vaccines, some are based on polysaccharides or sugars that form the outer coating of many bacteria to prevent bacterial infections. However, these do not elicit strong immune responses in infants; thus, their usefulness is limited. To overcome this limitation, studies have involved the attachment or conjugation of antigens to polysaccharides, known as conjugate vaccines, to improve the immune response. Conjugate vaccines protect against Haemophilus influenzae type b (Hib), pneumococcal, and meningococcal infections, even in young children. On the other hand, some vaccines are used to elicit immune responses against disease-causing proteins or toxins secreted by bacteria rather than against the pathogen. These antigens are chemically inactivated toxins, known as toxoids. Diphtheria and tetanus vaccines are representative toxoid vaccines.^143^

The use of nucleic acid-based vectors is an alternative method for whole-pathogen- or protein-based vaccination. Compared to conventional pathogen- or protein-based vaccines, nucleic acid-based vaccines are more stable, cost-effective, easy to manufacture, and safe to handle.^144,145^ The manufacturing process for nucleic acid vaccines is well established; therefore, it can be developed very quickly for emerging infectious diseases. DNA vaccines comprise a small circular piece of DNA called a plasmid that carries genes encoding particular proteins from the pathogen of interest. Moreover, messenger RNA (mRNA) is an intermediary between DNA and proteins and is used as a vaccine component. Encapsulated nucleic acids in nanoparticles can be used to deliver these vaccines to cells. Several experimental nucleic acid-based vaccines have been developed for SARS-CoV, H5N1, H1N1, Ebola, and Zika virus.^146^ Rather than delivering DNA or mRNA directly to cells, some vaccines use a harmless virus or bacterium as a vector or carrier to introduce genetic material into the cells. Several recombinant vector vaccines have been approved to protect animals from infectious diseases, including rabies and distemper.^147,148^ In particular, adenovirus-based vectors have been investigated for delivering the spike protein gene of SARS-CoV-2, as described in Table 2.

**Table 2:** The current COVID-19 vaccines under phase 3 clinical trials or FDA-approved for COVID-19. IM indicates intramuscular injection

Manufacturer	Type	Route
*Inactivated virus vaccines*		
Sinovac	Inactivated	IM
Wuhan Institute of Biological Products/Sinopharm	Inactivated	IM
Beijing Institute of Biological products/Sinopharm	Inactivated	IM
Bharat Biotech	Whole-Virion Inactivated	IM
*Non-replicating viral vector vaccines*		
University of Oxford/AstraZeneca	ChAdOx1-S^a^	IM
CanSino Biological Inc./Beijing Institute of Biotechnology	Adenovirus-based vector (type5)	IM
Gamaleya Research Institute	Adenovirus-based vector (rAd26-S + rAd5-S, type26 + type5)	IM
Janssen Pharmaceutical Companies	Adenovirus-based vector (type26)	IM
*Subunit vaccines*		
Novavax	Full length of recombinant SARS-CoV-2 glycoprotein nanoparticle vaccine with Matrix M(Adjuvant)	IM
*RNA vaccines*		
Moderna/NIAID	LNP-encapsulated mRNA^a^	IM
BioNTech/Fosun Pharma/Pfizer	LNP-encapsulated mRNA^a^	IM

^a^ indicates FDA-approved vaccines. (updated on the 23rd of December 2020; Adapt from WHO website^135^)

### Therapeutics technologies

Several types of drugs are available for the treatment of infectious diseases. Antibiotics, antivirals, and antifungals are powerful medicines for tackling infectious diseases caused by bacteria, viruses, and fungi, respectively. These drugs are used when the immune system is insufficient to overcome microbial infection. Because each drug has no cross-effectivity, these medicines are used for specific types of microbes.^149^

Several classes of antibiotics are available for the treatment of bacterial infections. Penicillin was the first discovered antibiotic, followed by other antibiotics, including cephalosporins, macrolides, tetracyclines, and quinolones, for the treatment of bacterial infections. These antibiotics can kill infectious bacteria and prevent their reproduction.^150^ Antiviral drugs are a class of medicines used to treat viral infections. Most antivirals attack particular targets and inhibit entry into host cells, replication, and assembly during their life cycle. Acyclovir, ribavirin, and oseltamivir (Tamiflu) are representative antiviral drugs.^151^ Antifungal reagents are drugs that selectively eliminate fungal pathogens in hosts with minimal toxicity to the host. Several types of antifungal drugs, such as polyene, azole, allylamine, morpholine, and antimetabolite antifungal drugs, are used to eliminate infectious fungi.^152^

Antibodies are important components of the immune system that fight infectious microbes. Serum containing antibodies taken from individuals who have recovered from infectious disease has been used for infection treatment since the early 20th century, and this serum therapy was gradually replaced by antibodies purified from pooled sera, intravenous immune globulin.^153,154^ Along with the development of the hybridoma method and monoclonal antibody (mAb) isolation for monoclonal antibody manufacturing technology, mAb is now considered a viable therapeutic modality for infectious disease targets,^153^ including newly emerging viral pathogens, such as Ebola,^155^ Zika,^156^ dengue,^157^ and COVID-19.^158,159^

Cell therapy is an alternative to the treatment of infectious diseases. Immune cells play a crucial role in controlling viral and fungal infections, and the goal is to eliminate pathogens through inflammatory reactions without collateral damage. They kill infectious microbes or modulate immune responses by communicating with adjacent immune cells through spurting cytokines, such as interleukins.^160^ The utilization of modified immune cells, such as chimeric antigen receptor-T (CAR-T) cells, is an attractive approach to conquer infectious diseases.^161^ CAR-T cells have the targeting specificity of a monoclonal antibody combined with the effector functions of cytotoxic T cells. They offer potential advantages over pathogen-specific T cells, such as antigen recognition independent of the MHC and the ability to be designed to specifically target the conserved and essential epitopes of the antigen, which allows them to overcome pathogen escape mechanisms. Some CAR-Ts have been designed to fight infectious diseases such as human immunodeficiency virus (HIV),^162^ hepatitis C virus (HCV),^163^ HBV,^164^ and cytomegalovirus (CMV).^165^

In addition, cell therapy using hMSCs is another approach for treating infectious diseases. hMSCs account for only 0.001–0.01% of the total population of nucleated cells is non-hematopoietic stem cells present within the bone marrow stromal compartment.^166^ hMSCs can differentiate into mesenchymal tissues, such as bone, cartilage, tendons, muscles, and adipose tissues. Multipotential stromal precursor cells can also differentiate into unrelated germline lineages via the trans-differentiation process.^167^ As the infusion of hMSCs into an injured region indicates the possibility of tissue regeneration and immune modulation, it is believed that they also have great potential in the treatment of infectious diseases. Based on these abilities, cell therapy using hMSCs is currently being investigated to develop treatments for tuberculosis,^168^ malaria,^169^ HIV,^170^ and, recently, COVID-19.^171^

### 3D bioprinted vaccines and drugs

Already being applied in tissue engineering, 3D bioprinting technology is also rapidly becoming a powerful method in pharmaceutical manufacturing and drug delivery. 3D printing technology allows the manufacturing of personalized medicine, which generally requires complex manufacturing methods due to individualized dosages and specific drug combinations. In this section, we describe the 3D bioprinting technologies for directly producing RNA vaccines and therapeutic drugs.

#### RNA printer (CureVac)

For the rapid formulation and delivery of vaccines, a proof-of-concept for the mRNA printing facility has been established for outbreak responses. The idea is that, following the design and validation of numerous RNA vaccines in parallel with the identification or sequencing of an emerging pathogen, the RNA printer would manufacture a clinic-ready vaccine. CureVac, a pioneer of mRNA printing systems, uses conventional manufacturing methods to develop mRNA vaccines against a range of viral infections, including yellow fever, Lassa fever, MERS, and COVID-19.^8,172,173^ Their original objective was to develop an automated or semi-automated robust system to deliver the company’s rabies vaccine.

#### Drug printer

The introduction of 3D printing to the pharmaceutical field was done in the hope of developing patient-centered dosages based on structural designs. The 3D printed tablets had controlled release properties that would reduce the frequency and number of dosages consumed by a patient as part of their daily routine. Reduced dosing frequency extended the release products and increased patient compliance for those who took the drug several times a day.^174^ That is, a patient can take one pill in the morning, not once every few hours or days.

Aprecia Pharmaceuticals reformulated the anti-epileptic medication levetiracetam, named Spritam.^175,176^ The first 3D printed tablets that disintegrate within seconds in an aqueous solution were developed through a proprietary powder bed and inkjet 3D printing technology known as ZipDose. Similar to selective laser sintering (SLS), the inkjet printing head drops a binding liquid onto a powdered layer. Depending on the size of the tablet, layers can be stacked up to 40 times, allowing for tighter packaging of the drug. In general, a single tablet containing 200 mg can contain 1000 mg, and this drug was approved by the FDA in 2015.^176^ The resulting pill is a high-dose medicine that is easy for people with epilepsy to swallow, break down in the body, and provide a steady dose over time.

Fina et al.^177^ prepared a paracetamol tablet using SLS with Kollicoat® IR or Eudragit® L100–55, which aided the sintering process using a Candurin® gold sheen. The printed tablet, called printlet, containing a theoretical value of drugs, showed good mechanical properties, including low friability (0.02–0.56%) and high crushing strength (284–485 N). In the Kollicoat®-based formulation, a pH-independent release characteristic with a release rate depending on the drug content was observed, whereas the Eudragit®-based formulation exhibited a pH-dependent release profile that was not correlated with drug loading.

Khaled et al.^178^ produced a polypill via 3D extrusion-based printing technology to treat diabetic patients suffering from hypertension.^179^ The polypill consists of a zero-order drug-based captopril osmotic pump compartment, joining layer, and sustained-release compartments of nifedipine and glipizide. When taking the pill, the joining layer disintegrates quickly; therefore, the polypill splits into a captopril compartment and a sustained-release compartment. 3D printing technology enables the regulation of independent release profiles by controlling the porosity for diffusion: one for the sustained release of ramipril, atenolol, and pravastatin, and the other for the immediate release of aspirin and hydrochlorothiazide. With this desired release profile in a tablet, patients with various risk factors, such as hypertension and dyslipidemia, can be treated simultaneously with a single tablet.

However, some patients require multiple medicines with more complex release profiles. Sun and Soh^180^ reported 3D printed customizable tablets that can accomplish any desired release profile. The tablet is comprised of three components: (1) a drug containing a surface-eroding polymer, (2) a drug-free surface-eroding polymer, and (3) an impermeable polymer that forms a protective coating. Constant, increasing, decreasing, and pulse release could be attained by designing various shapes of the surface-eroding polymer with drug compartments. These studies indicate that 3D printing technology has enormous potential for the manufacture of various multiple polypills in a tablet as well as for achieving complex and sophisticated drug release profiles. Although there remain many limitations in the 3D printing-combined pharmaceutical manufacturing process, these techniques are promising as an inexpensive and efficient way to create customized tablets.

### 3D bioprinted cell/drug delivery systems

Most chemotherapeutic drugs, including 5-fluorouracil, paclitaxel, and cisplatin, have low solubility; therefore, conventional medication techniques (e.g., intravenous injection and oral medication) cannot deliver the desired drug concentration to the diseased site. Worse, such drugs impair the entire body through systemic toxicity and cytokine release syndrome after infusion. Therefore, to overcome the aforementioned limitations of conventional drug delivery systems, various local treatment transfer systems containing small-molecule drugs, engineered cells, or vaccines to specific organs and cell types have been developed. Local therapeutic delivery systems aim to improve the effects of drugs against systemic side effects. Injectable or implantable systems with specific prolonged release kinetics and desired doses and geometries based on 3D printing technology are discussed in this section (Table 3).

**Table 3:** 3D-printed delivery systems

Target disease	Printing method	Printing material	Drug	Ref.
Bone infection	Extrusion	Hydroxyapatite nanocrystal-containing PCL	Rifampin	^181^
	Extrusion	Methacrylated hyaluronic acid and methacrylated gelatin-based hydrogels	Daptomycin	
Pancreatic tumor	Extrusion	PCL and poly(lactide-co-glycolide) (PLGA)	5-Fluorouracil	^20^
Osteomyelitis	Extrusion	PCL and PLGA	Tobramycin	^182^
Tumor	Extrusion	PLGA	Ovalbumin	^15^
Tumor	Stereolithography	E-Shell 300 3D (EnvisionTEC, biocompatible photopolymer)	Ovalbumin	^19^

#### Cell delivery systems

In addition to the development of cell-encapsulated scaffolds in tissue engineering studies, there are some macrophage- and antibiotic-laden delivery systems that reduce infection after surgery. Aldrich et al.^181^ presented a 3D-printed composite scaffold with antibacterial efficacy for treating bone infections after craniotomy. Antibiotic-laden PCL/hydroxyapatite constructs with hydroxyapatite-based hydrogel encapsulating macrophages were printed. The composite scaffolds were then implanted in a bone defect model with a *Staphylococcus aureus* craniotomy-associated biofilm. The biofilm dispersion was promoted by macrophages, which may secrete cytokines/chemokines that boost the antimicrobial activity of other glia/leukocytes related to the infection. The transformation of bacteria into a metabolically sensitive form was observed following antibiotic action. These results indicate that the treatment of established biofilms could be a beneficial alternative to antibiotic therapeutics.

#### Sustained drug release systems

Antibiotic drug-loaded systems are also required after operations on internal organs. The polymeric patch developed by Yi et al.^20^ was fabricated with various geometries of the administration site using an extrusion-based printing system. The patch, made of PCL and poly(lactide-co-glycolide) (PLGA) intermixed with 5-fluorouracil, enabled sustained drug release over four weeks in a rabbit model, leading to a significantly smaller pancreatic tumor. Shim et al.^182^ presented tobramycin-laden 3D-printed scaffolds for treating osteomyelitis. Tobramycin showed thermal stability for extrusion of the polymer blend with PCL and PLGA during the 3D printing process, and the sustained release profile in vitro could be observed for up to 51 days. After 8 weeks of in vivo implantation using an *S. aureus*-infected rat model, reduction in inflammation of the femur and new bone formation were observed, while inflammation and phagocytosis were observed in the vicinity of the implantation sites in the no implantation group. These studies have utilized biodegradable polymers (e.g., PCL and PLGA) to release the drugs. These polymers have backbone ester bonds that can be swollen and then hydrolyzed by water. Hydrolytic degradation also occurs in an in vivo environment by body fluids, and the drug is released simultaneously. The release rate of drugs can be regulated by controlling the hydrolysis rate of each polymer. Therefore, it can be noted that 3D printed scaffolds show promise in personalized medicine because of their ability to rapidly build custom scaffolds specific to the shape of the diseased defect.

#### Pulsatile drug delivery systems

Some therapeutics demand specific kinetics release and biodistribution. In 2017, McHugh et al.^15^ introduced a single injection platform for pulsatile release of vaccines. Ovalbumin was encapsulated in micro-PLGA-capsules via 3D printing technology, and the capsules were delivered by subcutaneous injection in mice. The release profile was regulated by controlling the ratio of lactide to glycolide, which adjusted the degradation rate. Based on these approaches, an injectable system containing multiple polymeric particles with different degradation rates could be delivered together in a single dose at the initial treatment time, allowing the design of a release profile consistent with conventional vaccination points over time.

#### Aerosolized delivery systems

In addition to local direct delivery at the surgical site, noninvasive mucosal delivery of biologics has been developed in parallel. Aran et al.^19^ developed a 3D-printed device named MucoJet for oral vaccination. MucoJet was designed to deliver fluorescein-labeled ovalbumin via a high-pressure liquid jet across the buccal mucosa and 3D-printed using biocompatible and water-resistant photopolymerizable plastic resin. It contains the external and inner compartments, and the compartments have propellant and vaccine reservoirs, respectively. The MucoJet device was also sealed with a polymeric membrane valve. When the polymeric membrane is exposed and dissolves, water within the exterior compartment contacts the chemical propellant in the propellant reservoir, generating CO_2_ gas. The propellant chamber’s increased pressure pushes the piston toward the vaccine reservoir, allowing the inner chamber to eject the vaccine. Ex vivo application showed that MucoJet increased delivery efficiency by approximately eightfold compared to conventional dropwise treatments. Furthermore, the specific antibody titers of IgG and IgA using blood and tissue after application in vivo showed that the immunogenicity of buccally administered antigens was dramatically enhanced by three orders of magnitude compared to that of drop-wise treatments.

## Conclusions and future outlook

The COVID-19 pandemic has offered lessons on the necessity of a fast response to appropriately deal with the emergence and transmission of new infectious diseases. This worldwide crisis has generated considerable attention in relation to the need for rapid development of vaccines and therapeutics to prevent the spread of pathogens. As the conventional drug development pipeline includes extremely time-consuming and expensive processes, novel platform technology is required to retool the pipeline to accelerate drug discovery.

Cutting-edge biofabrication technologies have shown potential for improving the preclinical drug development stages. Organoids and microfluidic organs-on-chips have been developed to reproduce the pathophysiological features of diseased tissues and to elucidate the underlying mechanism of disease development and to test candidate drugs. In addition, 3D bioprinting has displayed creating in vitro models of various tissues that can be applied for infectious disease research. As 3D bioprinting is superior in the construction of complex 3D structures with multiple materials, this technology has been widely used to create primary organs in vitro. The 3D bioprinted organs have shown not only similarities to the anatomy of the corresponding organs but also to highly mature biological functions, which is the most important aspect in understanding infectious diseases.

However, the high-throughput production of reliable in vitro organ models in many wells of conventional microplates is a critical requirement in pharmaceutical and medical applications. In this regard, improvement of the resolution and productivity of 3D bioprinting is an essential challenge.^134,183^ As inkjet bioprinting shows high resolution in the <30 μm range, droplet-based cellular constructs have been produced in the form of arrays in a small area, such as a slide glass, for application to high-throughput drug screening.^134,184^ The bioprinted droplets exhibit combinations of different cells or double- or triple-layered structures.^134,184,185^ In addition, laser-assisted bioprinting with DLP demonstrated 2D patterning with different cell types in wells of a 96-well plate, while 3D complex constructs were constructed as well.^186^ These remarkable improvements in the resolution of 3D bioprinting suggest the possibility of using this technology in the field of drug screening and new drug development. However, high-resolution bioprinting should produce functional tissue formation to achieve meaningful results in the study of diseases and drugs.

In the design and production of medicine, the recent technologies of vaccines (e.g., nucleic acid-based vectors) and therapeutics (recombinant antibodies) have presented the possibility of rapid treatment development against new pathogen emergence. Moreover, the convergence of vaccine/therapeutic technologies with the 3D bioprinting of medicine and/or drug delivery systems could have a considerable impact on the advancement of the drug development process. Direct 3D printing of drugs has been in the spotlight because of the customization efficiency of the printing process. As 3D printing allows the combination of different materials on-demand, personalized drug compositions or doses in a tablet can be realized by controlling the printing process. In addition, the integration of medicine with biomaterials has enabled the creation of diverse delivery systems to increase the efficiency of drug administration. However, 3D bioprinting of medicine and delivery systems is still in its infancy; thus, many studies are required to develop more applicable manufacturing technologies for vaccines and therapeutics.^25,187^

As 3D bioprinting is based on a computational process from modeling to manufacturing, this technology is highly favorable for automation.^188,189^ With the recent development of artificial intelligence and autonomous control technologies, the automation of 3D printing has begun to be investigated.^190^ If 3D bioprinting can be integrated with automation technology, 3D bioprinting-based approaches for producing in vitro models and medicines will be embraced in the pharmaceutical industry to speed up the retooling process. Likewise, 3D bioprinting is expected to offer new technologies for better control of infectious diseases and for faster drug discovery in future medicine.
